# A Modified Magnetic Gradient Contraction Based Method for Ferromagnetic Target Localization

**DOI:** 10.3390/s16122168

**Published:** 2016-12-17

**Authors:** Chen Wang, Xiaojuan Zhang, Xiaodong Qu, Xiao Pan, Guangyou Fang, Luzhao Chen

**Affiliations:** 1 Key Laboratory of Electromagnetic Radiation and Sensing Technology, Chinese Academy of Sciences, Beijing 100190, China; dongdongqu@126.com (X.Q.); xiaopan13@mails.ucas.ac.cn (X.P.); gyfang@mail.ie.ac.cn (G.F.); chluzh@163.com (L.C.); 2 University of Chinese Academy of Sciences, Beijing 100039, China

**Keywords:** ferromagnetic target localization, magnetic gradient tensor, magnetic dipole, iterative algorithm

## Abstract

The Scalar Triangulation and Ranging (STAR) method, which is based upon the unique properties of magnetic gradient contraction, is a high real-time ferromagnetic target localization method. Only one measurement point is required in the STAR method and it is not sensitive to changes in sensing platform orientation. However, the localization accuracy of the method is limited by the asphericity errors and the inaccurate value of position leads to larger errors in the estimation of magnetic moment. To improve the localization accuracy, a modified STAR method is proposed. In the proposed method, the asphericity errors of the traditional STAR method are compensated with an iterative algorithm. The proposed method has a fast convergence rate which meets the requirement of high real-time localization. Simulations and field experiments have been done to evaluate the performance of the proposed method. The results indicate that target parameters estimated by the modified STAR method are more accurate than the traditional STAR method.

## 1. Introduction

A ferromagnetic target generates a measurable magnetic anomaly under the geomagnetic field and can be adequately modeled at a distance by an equivalent magnetic dipole moment [1,2]. Magnetic anomaly signals can be used to invert the target parameters—i.e., position and magnetic moment—which have many applications such as unexploded ordnance detection [3,4,5], underwater magnetic tracking [6,7], intruder detection [8], biomedical applications [9], and indoor localization [10]. Usually magnetic anomaly signals are much weaker than the geomagnetic field intensity and they cannot be measured by magnetometers directly. Gradiometers can eliminate the interference of geomagnetic fields and are widely used in magnetic anomaly detection.

The full characterization of a point dipole source requires the estimation of six parameters, three describing the target location and three describing its magnetic moments [1]. Therefore, it requires at least six equations to estimate target parameters. The analytical method described in [11] estimates two unit vectors representing the bearing vector and the magnetic moment orientation vector with five equations of magnetic gradient tensor. The distance and the magnetic moment magnitude cannot be calculated with this method. In addition, there are four solutions for bearing vector and unit magnetic moment vector. To solve the problem of multiple solutions, an improved method is proposed by Wynn et al. [12]. The host platform is assumed to move in a straight line and the speed is known, then a unique solution can be obtained by the improved method. However, influenced by actual terrain, platform motion is difficult to be paralleled to straight line. Moreover, the target is assumed static in this method. Therefore, it is not applicable to moving-target localization. 

In recent years, a closed-form localization formula was proposed in [13], in which the position is calculated by multiplying the inverse of magnetic gradient tensor with the magnetic field generated by the target. Based on [13], a novel localization method with higher accuracy is proposed by Nara et al. By transforming Euler’s equation into an integral form, the analytical solution with the surface integrals of magnetic flux is proposed [14]. The dipole position can be inverted directly with the analytical solutions in these methods. The high real-time characteristic makes them more applicable to tracking magnetic sources. However, these methods require the measurements of the magnetic anomaly field. It is very difficult to separate the magnetic field generated by a ferromagnetic target from the geomagnetic field. Therefore, these methods are not applicable to a ferromagnetic target.

The numerical inversion methods give another means of magnetic localization. Barrell et al. [15] and Vaizer et al. [16] estimate the magnetic target parameters with linear statistical analysis. Brisan et al. introduces a particle filter to magnetic target tracking [1]. Liu et al. introduces an improved particle swarm optimization algorithm for ferromagnetic target localization [17]. The results of this statistical recursive method are more robust against the noise. However, different measurement points are required in non-linear numerical inversion methods, which lead to low real-time characteristics.

In order to locate a ferromagnetic target with a single measurement point, Wiegert puts forward a Scalar Triangulation and Ranging (STAR) method [18]. The STAR concept for localization of ferromagnetic target is based upon the unique properties of “total” magnetic gradient contraction $C_{T}$ which is defined as the matrix norm of the magnetic gradient tensor. The magnetic gradient contraction $C_{T}$ is a rotationally invariant and robust scalar that is not affected by changes in sensing platform orientation. Therefore, this method can be used in high-mobility sensing platforms. However, the contour of $C_{T}$ is assumed as a sphere, which actually is an ellipsoid. Therefore, the results of localization have inherent errors called “asphericity errors” which have a great influence on the estimation of the bearing vector [18]. Moreover, the inaccurate value of the bearing vector leads to larger errors in the estimation of the magnetic moment vector. Although Wiegert proposes an improved method to correct the estimated errors of the distance between the object and the sensor array, the asphericity errors are still not corrected [19]. Sui also proposes a method to correct the STAR method [20]. However, this method needs a very high signal-to-noise ratio (200~400). In addition, this paper has no experiment results. Actually, it is very difficult to meet the need of high signal-to-noise ratio in field experiments.

In this paper, a modified STAR method is proposed for ferromagnetic target localization. It compensates for the asphericity errors of traditional STAR method with an iterative algorithm. The parameters of a ferromagnetic target can be accurately estimated by this method. The proposed method has a fast convergence rate which meets the requirement of high real-time localization. Simulations and field experiments have been done to test the performance of the proposed method. The results show that the modified STAR method is more accurate in ferromagnetic target localization than the traditional STAR method.

## 2. STAR Concept and Asphericity Errors 

When the distance between the ferromagnetic target and the sensor array is more than three times the physical dimensions of the target, the magnetic signal is expressed as follows:

(1) $$B=\frac{\mu_{0}}{4\pi }\frac{3\left(\vec{M}\cdot \vec{R}\right)\cdot \vec{R}-\vec{M}\cdot r^{2}}{r^{5}}$$

where $\mu_{0}$ represents the permeability of vacuum. $\vec{R}$ represents the position vector. r represents the magnitude of $\vec{R}$. $\vec{M}$ represents the magnetic moment vector of target. The magnetic gradient tensor expressions are expressed as follows:

(2) $$G=\left[\begin{array}{ccc} \frac{\partial B_{x}}{\partial x} & \frac{\partial B_{x}}{\partial y} & \frac{\partial B_{x}}{\partial z}\\ \frac{\partial B_{y}}{\partial x} & \frac{\partial B_{y}}{\partial y} & \frac{\partial B_{y}}{\partial z}\\ \frac{\partial B_{z}}{\partial x} & \frac{\partial B_{y}}{\partial y} & \frac{\partial B_{z}}{\partial z} \end{array}\right]=\left[\begin{array}{ccc} B_{xx} & B_{xy} & B_{xz}\\ B_{yx} & B_{yy} & B_{yz}\\ B_{zx} & B_{zy} & B_{zz} \end{array}\right]$$

The element in tensor is expressed as follows:

(3) $$G_{ij}=\frac{\partial B_{i}}{\partial j}=-3\frac{\mu_{0}}{4\pi }\frac{\left(\vec{M}\cdot \vec{R}\right)\left(5r_{i}r_{j}-r^{2}\delta_{ij}\right)-r^{2}\left(r_{i}M_{j}-r_{j}M_{i}\right)}{r^{7}}$$

where i,j=x,y,z. As a result of Maxwell’s Equations, the gradient tensor matrix is traceless and symmetric. Thus, measurement of just five independent tensor components is sufficient to determine the full, nine-component magnetic gradient tensor [18]. The magnetic gradient contraction $C_{T}$ is defined as the matrix norm of the magnetic gradient tensor.

(4) $$\begin{array}{c} C_{T}^{2}={\sum \left(G_{ij}\right)}^{2}={\left(\partial B_{x}/\partial x\right)}^{2}+{\left(\partial B_{x}/\partial y\right)}^{2}+{\left(\partial B_{x}/\partial x\right)}^{2}\\ +{\left(\partial B_{x}/\partial z\right)}^{2}+{\left(\partial B_{y}/\partial x\right)}^{2}+{\left(\partial B_{y}/\partial y\right)}^{2}+{\left(\partial B_{y}/\partial z\right)}^{2}\\ +{\left(\partial B_{z}/\partial x\right)}^{2}+{\left(\partial B_{z}/\partial y\right)}^{2}+{\left(\partial B_{z}/\partial z\right)}^{2}\\ C_{T}=\sqrt{C_{T}^{2}}=k\frac{\mu_{0}}{4\pi }\frac{\left|\vec{M}\right|}{r^{4}} \end{array}$$

where *k* is an asphericity parameter, which characterizes the departure of the $C_{T}$ field from perfect spherical symmetry. Actually, *k* is a number that varies from about 7.3 for “polar” points aligned with the dipole axis to 4.2 for points on the “equator” transverse to the dipole axis [18]. Conversely, for contours of $C_{T}$, the ratio of the polar diameter to a diameter on the equator is about 1.14 to 1. In accordance with Equation (4), if k≈constant, then the different values of $C_{T}$ are only related to different distances. The relationship is expressed as follows:

(5) $$\frac{C_{T1}}{C_{T2}}=\frac{r_{2}^{4}}{r_{1}^{4}}$$

where $C_{T1}$ and $C_{T2}$ are the magnetic gradient contractions measured simultaneously in points 1 and 2. Points 1 and 2 are at distances $r_{1}$ and $r_{2}$($r_{2}=r_{1}+\Delta r$) from the magnetic object. The distance between the target and sensor array can be triangulated by:

(6) $$\begin{array}{c} \frac{C_{T1}}{C_{T2}}=\frac{{\left(r_{1}+\Delta r\right)}^{4}}{r_{1}^{4}}\\ r_{1}=\Delta r{\left[{\left(C_{T1}/C_{T2}\right)}^{0.25}-1\right]}^{-1} \end{array}$$

**Figure 1:**
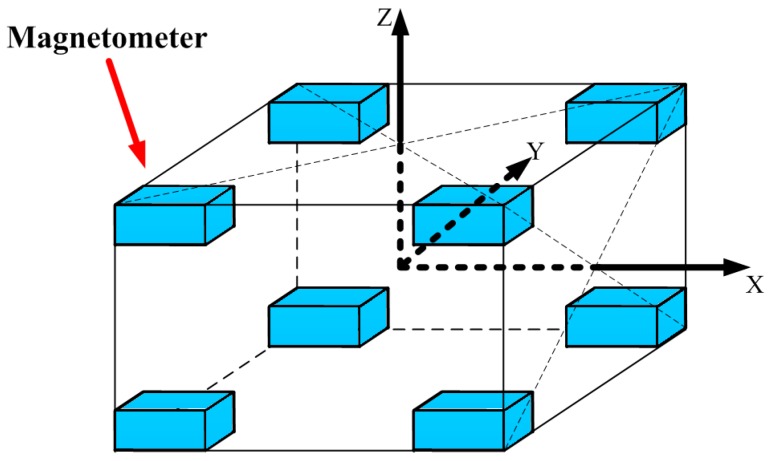
Structure of the magnetometer array to implement the STAR method.

The contour of the magnetic gradient contraction is a sphere when *k* is assumed as a constant. Therefore, its gradient points to the center of sphere. In order to implement the STAR concept, a cubic array of eight tri-axial fluxgate magnetometers is designed, as shown in Figure 1. 

The magnetometers are put on the eight vertices of the cube, and the magnetic tensors of six faces are measured. $C_{T}$ of each face is calculated by Equation (4) and $C_{\mathrm{TX}+}$, $C_{\mathrm{TX}-}$, $C_{\mathrm{TY}+}$, $C_{\mathrm{TY}-}$, $C_{\mathrm{TZ}+}$, $C_{\mathrm{TZ}-}$ are obtained. The gradient of $C_{T}$ is expressed as follows:

(7) $$\nabla C_{T}=\frac{C_{\mathrm{TX}+}-C_{\mathrm{TX}-}}{dx}i+\frac{C_{\mathrm{TY}+}-C_{\mathrm{TY}-}}{dy}j+\frac{C_{\mathrm{TZ}+}-C_{\mathrm{TZ}-}}{dz}k$$

where, dx, dy and dz represent the baseline of each axis, respectively. The unit bearing vector is expressed as:

(8) $$\vec{r_{0}}\approx \frac{\nabla C_{T}}{\left|\nabla C_{T}\right|}$$

The distance between target and the measurement point can be derived by Equation (6) and the expression is written as follows:

(9) $$r=\{\begin{array}{c} \Delta S_{X}\{{\left[{\left(C_{TX-}/C_{TX+}\right)}^{0.25}-1\right]}^{-1}+0.5\}\\ \Delta S_{Y}\{{\left[{\left(C_{TY-}/C_{TY+}\right)}^{0.25}-1\right]}^{-1}+0.5\}\\ \Delta S_{Z}\{{\left[{\left(C_{TZ-}/C_{TZ+}\right)}^{0.25}-1\right]}^{-1}+0.5\} \end{array}$$

where $\Delta S_{X}=\left(\begin{array}{ccc} dx & 0 & 0 \end{array}\right)\cdot \vec{r_{0}},\;\Delta S_{Y}=\left(\begin{array}{ccc} dy & 0 & 0 \end{array}\right)\cdot \vec{r_{0}},\;\Delta S_{Z}=\left(\begin{array}{ccc} dz & 0 & 0 \end{array}\right)\cdot \vec{r_{0}}$ represent the projection of the X, Y, Z baseline on the bearing vector, respectively. Theoretically, the method needs the projection on only one of three baselines (X, Y, Z). Finally, the position vector is calculated by the follow expression.

(10) $$\vec{R}=r\cdot \vec{r_{0}}$$

Magnetic moment vector $\vec{M}$ is calculated by putting position vector $\vec{R}$ into Equation (3).

In STAR method, the expressions of position vector are derived on the assumption that *k* is a constant. The asphericity parameter *k* will be essentially constant over the volume of a sensor system when r>3 times the distances between $C_{T}$ measurement points in the sensor system. Therefore, the errors caused by asphericity parameter *k* in Equation (9) are very small. However, the bearing vector calculated by Equation (8) is much more sensitive to the asphericity parameter. Even neglecting the sensors noise, the estimated parameters with STAR method still have errors. Thus, differences between the true values of target parameters and the respective estimated values are inherent to the STAR method.

## 3. Asphericity Errors Compensation Algorithm

The parameter k is derived by Equation (4) and rewritten as follows:

(11) $$\begin{array}{c} k=3\sqrt{4\cos^{2}\theta +2}\\ \cos\theta =\vec{m_{0}}\cdot \vec{r_{0}} \end{array}$$

where $\vec{m_{0}}$ represents the unit magnetic moment vector. The expression of the magnetic gradient contraction $C_{T}$ is derived by putting Equation (11) into Equation (4).

(12) $$C_{T}=3\sqrt{4{\left(\vec{m_{0}}\cdot \vec{r_{0}}\right)}^{2}+2}\cdot \frac{\mu_{0}}{4\pi }\frac{\left|\vec{M}\right|}{r^{4}}$$

Then the gradient of $C_{T}$ is derived and expressed as follows.

(13) $$\nabla C_{T}=\frac{3\mu_{0}}{4\pi }\left(\sqrt{4{\left(\vec{m_{0}}\cdot \vec{r_{0}}\right)}^{2}+2}\cdot \nabla \left(\frac{\left|\vec{M}\right|}{r^{4}}\right)+\frac{\left|\vec{M}\right|}{r^{4}}\cdot \nabla \left(\sqrt{4{\left(\vec{m_{0}}\cdot \vec{r_{0}}\right)}^{2}+2}\right)\right)$$

Simplify Equation (13) and the final expression of $\nabla C_{T}$ is expressed as follows:

(14) $$\nabla C_{T}=-\left(3\sqrt{4\cos^{2}\theta +2}+\frac{12\cos^{2}\theta }{\sqrt{4\cos^{2}\theta +2}}\right)\cdot \frac{\left|\vec{M}\right|}{r^{5}}\vec{r_{0}}+\frac{12\cos\theta }{\sqrt{4\cos^{2}\theta +2}}\cdot \frac{\left|\vec{M}\right|}{r^{5}}\vec{m_{0}}$$

It is shown that the asphericity errors of STAR are caused by the second item in Equation (14). The value of position vector could be calculated accurately as long as the second item is known. The magnetic moment vector estimated by Equation (3) has low accuracy due to the asphericity errors of the STAR method. Therefore, an iteration algorithm is proposed to update the magnetic moment vector and position vector with Equations (3) and (14). Due to magnetometer errors, misalignment of magnetometer array, and the distortion field, the measurement errors of magnetic gradient tensor may reach hundreds of nanoteslas [21,22,23]. Even after calibration, the residual errors of measurements are at least several nanoteslas and disturb the localization results. Meanwhile, the magnetic moment vector estimated by STAR method is not stable. Therefore, the convergence rate will be slow if Equation (14) is used directly in an iteration algorithm. To solve the above problem, a unit vector is defined as follows:

(15) $$\vec{V}=\left(C_{1}\vec{r_{0}}+C_{2}\vec{m_{0}}\right)/\sqrt{C_{1}^{2}+C_{2}^{2}+2C_{1}C_{2}\cos\theta }=\frac{\nabla C_{T}}{\left|\nabla C_{T}\right|}$$

where,

(16) $$\begin{array}{c} C_{1}=3\sqrt{4\cos^{2}\theta +2}+\frac{12\cos^{2}\theta }{\sqrt{4\cos^{2}\theta +2}}\\ C_{2}=\frac{12\cos\theta }{\sqrt{4\cos^{2}\theta +2}} \end{array}$$

The algorithm is performed as follows:

Step 1: The initial value of position vector $\vec{R}$ is estimated by STAR method.
Step 2: Substituting position vector $\vec{R}$ into Equation (3), the magnetic moment vector $\vec{M}$ is estimated by least square method.
Step 3: The parameters $C_{1}$ and $C_{2}$ in Equation (16) are calculated with the estimated values of $\vec{R}$ and $\vec{M}$.
Step 4: The new unit bearing vector is calculated by the following expression.
  (17) $$\vec{r_{\mathrm{new}}}=\frac{\sqrt{C_{1}^{2}+C_{2}^{2}+2C_{1}C_{2}\cos\theta }\cdot \vec{V}-C_{2}\vec{m_{0}}}{C_{1}}$$

Go back to Step 2 and calculate the new magnetic moment vector until the position vector meets the condition of convergence. There are two kinds of convergence conditions. One is that the difference between two adjacent iterations is less than a specified value. The other one is that the iteration reaches the limit.

## 4. Simulation Study

In order to estimate theoretical accuracy of these two algorithms, the STAR method and the proposed method, a set of synthetic data was made. The magnetometer array was shown in Figure 1. The baselines of X, Y, and Z were all 300 mm. The trajectory of the magnetic target was shown as follows:

(18) $$\begin{array}{cc} \begin{array}{c} x=4\cos(\theta )m\\ y=4\cos(\theta )m\\ z=-3m \end{array} & \theta \in \left(0∼2\pi \right) \end{array}$$

**Figure 2:**
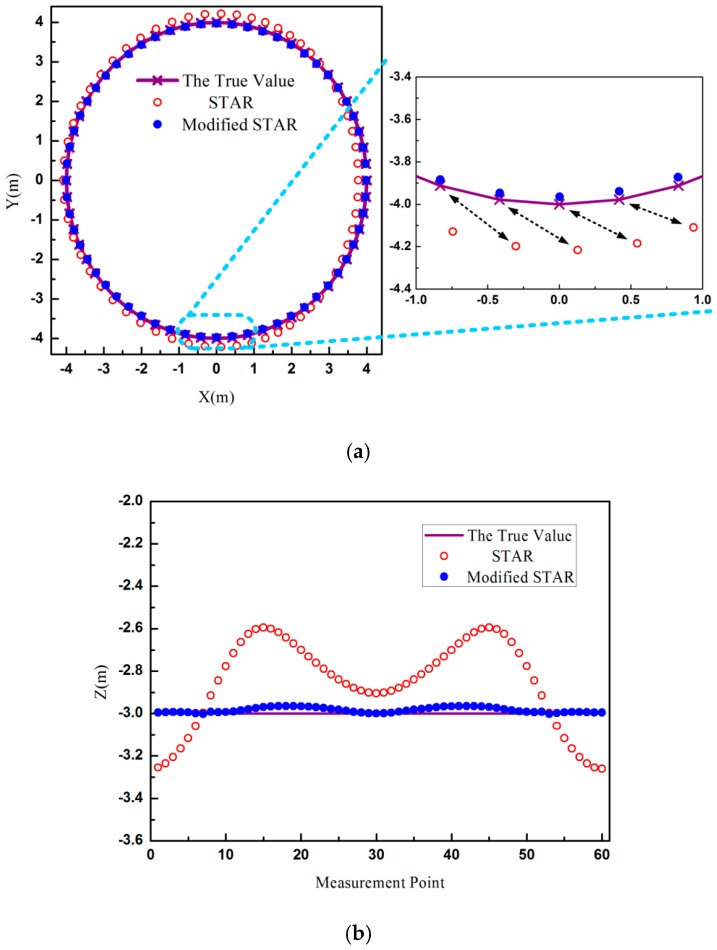
The estimated positions of two methods. (**a**) The estimated positions on the XY plane; (**b**) The estimated positions on the Z-axis.

The magnetic moment vector was (100, 0, 100) Am^2^. In the first simulation experiment, the root mean square (RMS) of magnetometers noise was 0.05 nT. The simulation results were shown in Figure 2.

It was shown that the localization accuracy of STAR method was much lower. In the amplified picture of Figure 2a, the positions calculated by STAR method were on the bottom right of the true value, as indicated by the arrows. The X-axis differences between the true value and the estimated value of STAR were even more than 0.5 m, and the Y-axis differences reached to 0.37 m. As shown in Figure 2b, the maximum error of STAR method in Z-axis was about 0.4 m. Meanwhile, the localization errors of STAR were closely related with the measurement point. Compared with STAR method, the proposed method could compensate almost all asphericity errors. The positions calculated by the modified STAR method almost overlapped the true values. The maximum errors of the proposed methods on X-axis, Y-axis, and Z-axis were 3 cm, 4 cm, and 3 cm, respectively. Considering the distance between magnetic target and magnetometer array, the errors of localization of the proposed method were less than 1%. In this simulation experiment, the magnetic moment was very large, and the signal-to-noise ratio (SNR) was more than 40 dB. Therefore, the noises of the magnetometers had little effect on the localization results. The errors of the modified STAR method were mainly from two parts. One was the tensor measurement errors caused by using the difference between magnetometers to approximate the magnetic gradient field. The other one was the errors caused by the asphericity parameter *k* and the geometry approximation when Equation (9) was used to calculate the distance. 

**Figure 3:**
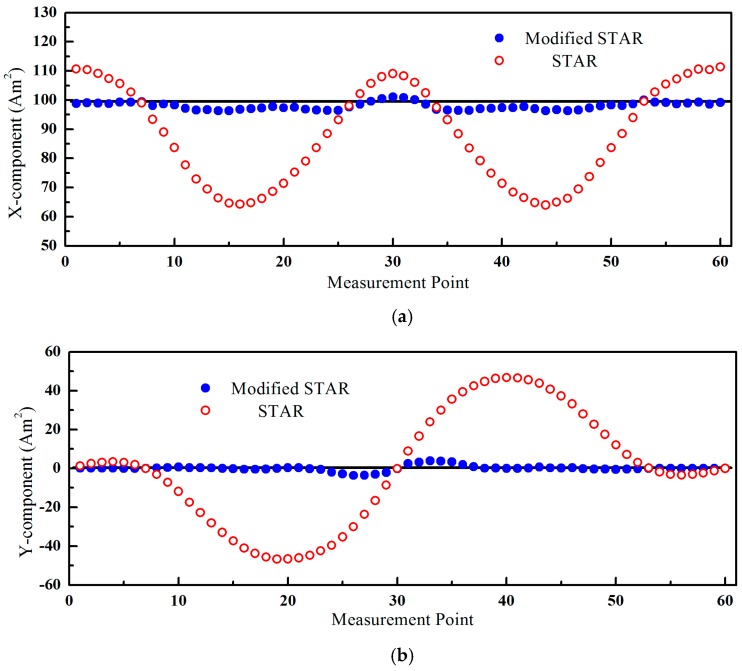

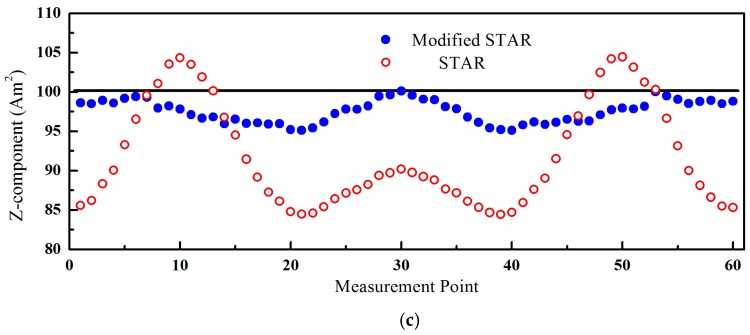
The estimated magnetic moment of the two methods. (**a**) X-component; (**b**) Y-component; (**c**) Z-component.

The magnetic moments estimated by these two methods were shown in Figure 3. The asphericity errors had a greater influence and the errors of STAR were more than 45 Am^2^, shown as Figure 3b. The estimated values of the modified STAR method were consistent with the true values.

**Figure 4:**
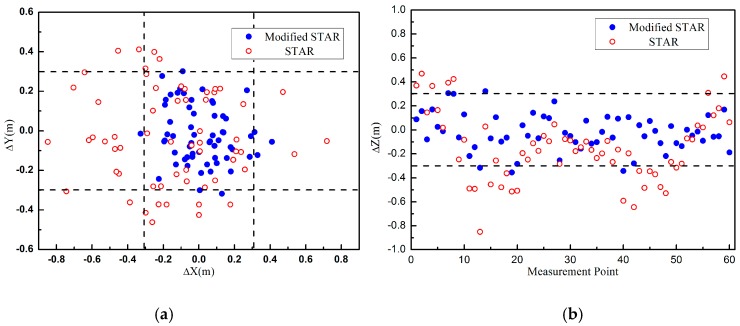
The error distributions of these two methods: (**a**) The error distributions of these two methods on XY plane; (**b**) The error distributions of these two methods in Z-axis.

Actually, the measurement errors of magnetic gradient tensors included three parts, the noise of magnetometers, errors of difference approximation, and the residual errors after the array system calibration process. Usually, the residual errors were much bigger than the others. So in the second experiment, the measurements of magnetic gradient tensors were added Gaussian white noise with different SNR to simulate the residual errors. The other simulation parameters were unchanged. When the SNR was about 20 dB, the localization error distributions of these two methods were shown in Figure 4. Similar to the first experiment, the accuracy of the proposed method was higher than the traditional STAR method. The errors of the proposed method were mainly caused by the residual errors and the errors of the STAR method were caused by both the residual errors and the asphericity errors.

**Figure 5:**
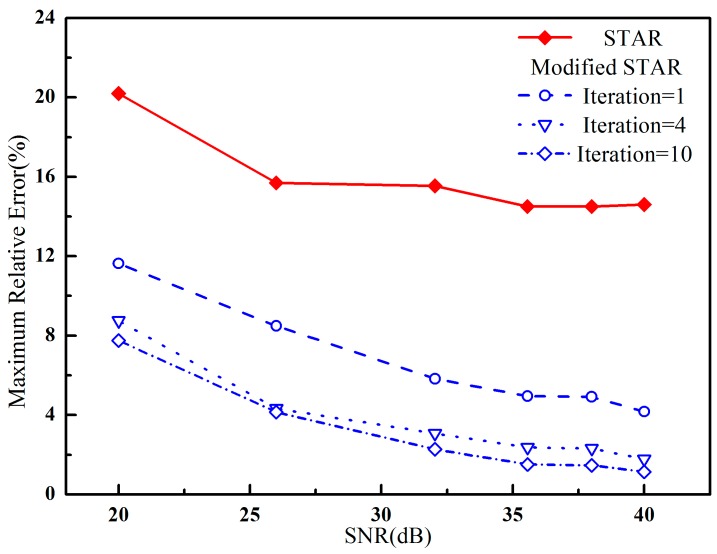
The maximum relative error between the estimated parameters and the true values versus SNR using synthetic data with additive Gaussian noise.

In this simulation experiment, white Gaussian noise was added to the synthetic data with different SNR. We have focused on the errors between the estimated parameters and the true values with different iterations. The maximum relative error with different iterations versus SNR was presented in Figure 5. It was shown that the accuracy of the proposed method was much higher than that of the STAR method. The relative error of the modified STAR method decreased as the iteration number increased. Meanwhile, the accuracy almost reached to the theoretical limit when the iteration number was up to 4. Therefore, the convergent rate is fast.

## 5. Experiment Result

**Figure 6:**
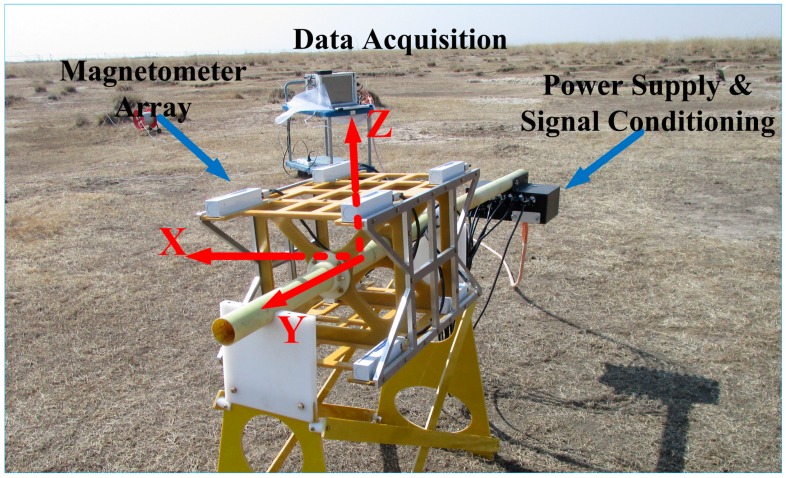
The experimental system.

To evaluate the proposed method, a field experiment was carried out in Hebei province, China. The experiment system consisted of a magnetometer array, a module of power supply and signal conditioning, and a module of data acquisition, as shown in Figure 6. The magnetometer array contained eight tri-axial fluxgate magnetometers mounted on the vertices of the cube. The baselines of the X-axis, Y-axis, and Z-axis were 300 mm, 400 mm, and 300 mm, respectively. The model numbers and manufacturers of the magnetometer and data acquisition were shown as Table 1. After calibration, the standard deviations of the magnetic gradient tensor measurements were about 2 nT.

**Figure 7:**
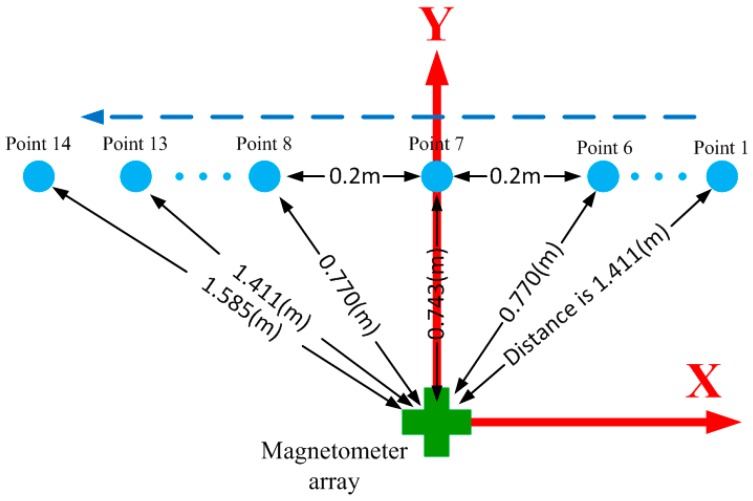
The diagram of the object trajectory.

The ferromagnetic object in the localization experiment was an iron pipe whose diameter and height were 10 and 20 cm, respectively. The ground of the experimental region was not very flat, so a long board was used to ensure the height difference between object and the sensor array remains unchanged. The iron pipe was upright during the whole experiment and the trajectory was a line parallel with the X-axis. The distance between each measurement point was 20 cm, as shown in Figure 7. The object positions with regard to the array reference coordinates were measured by tape and trajectory was expressed as follows:

(19) $$\begin{array}{cc} \begin{array}{l} x=1.4-0.2N\\ y=0.5\\ z=-0.55 \end{array} & \left(N=1,2,\cdots ,14\right) \end{array}$$

**Figure 8:**
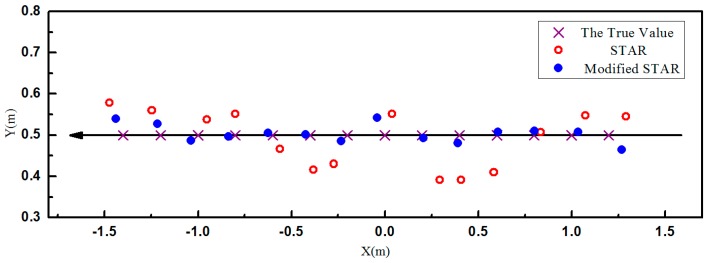
The estimated position of test specimen on the XY plane.

**Figure 9:**
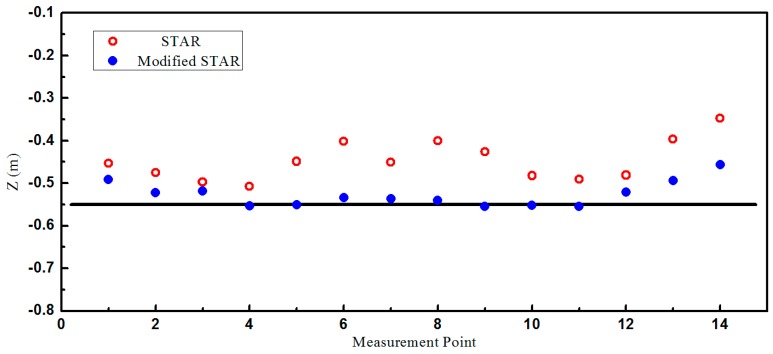
The estimated position of test specimen on the Z-axis.

The localization results were shown in Figure 8 and Figure 9. As predicted by the simulations, the accuracy of the proposed method was much higher than the traditional STAR method. It is observed that the localization errors of the traditional STAR method were at the largest when x=±0.5. It was probably because that the asphericity errors had great effect on the localization accuracy. The localization errors of the proposed method were much smaller because the asphericity errors had been compensated. It is observed that the accuracy of the point x=0 is less than the adjacent points. It was probably because errors caused by using the difference between magnetometers to approximate the magnetic gradient field became much larger when the object was very close to the array. The RMS errors of these two methods in X, Y, and Z were shown in Table 2. The smaller RMS localization errors showed that the modified STAR method was more accurate and stable than the traditional STAR method.

We had examined the computation times of the traditional STAR method and the proposed method which were performed in LabVIEW. Timings were taken on a PC with a dual-core Intel processor that ran at a nominal clock speed of 2.4 GHz. The computation times were shown in Table 3.

It was shown that the computation time of the proposed method is about twice the traditional STAR method, but 6.25 ms (around 160 Hz) is still considered acceptable for such real-time applications. The proposed method is able to increase the accuracy without sacrificing much on the real-time performance.

**Figure 10:**
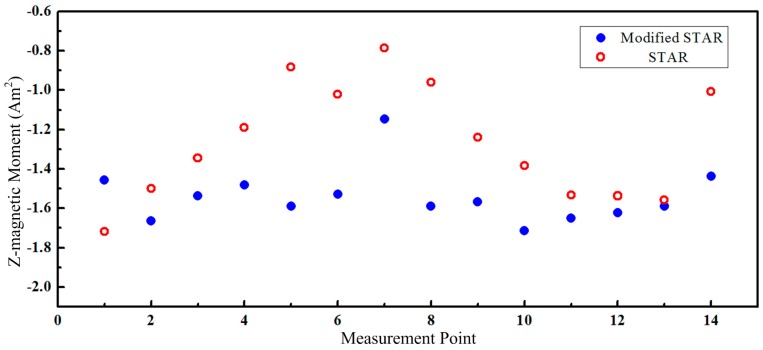
The estimated Z-magnetic moment of test specimen.

The estimated Z-magnetic moment with these two methods was shown in Figure 10. The estimated values using the proposed model were more consistent. Only small changes were observed when the pipe moved closer to the magnetometer array. The estimated results for other two components were similar. The mean value and RMS error of the estimated magnetic parameters were shown in Table 4. Therefore, the estimated magnetic moments with modified STAR method were more reliable.

## 6. Conclusions

In this work, we have investigated a modified STAR method for ferromagnetic target localization. The asphericity errors of the traditional STAR method are compensated by an iteration algorithm. Consequently, the accuracy of the estimated position vector and magnetic moment vector are significantly improved. Almost all asphericity errors can be compensated with only four iterations. Meanwhile, the experiments show that the proposed method is able to increase the accuracy without sacrificing much on the real-time performance. The field experimental results show that the localization accuracy of the proposed method is much higher than the traditional STAR method. As for the magnetic parameters estimation, the results of this method are more stable. The positive outcomes indicate that the modified STAR method can be a potential candidate for ferromagnetic target localization.

## Figures and Tables

**Table 1:** The Specification of the Magnetometer and Data Acquisition.

Hardware	Manufacturer	Model Number
Magnetometer	Bartington	Mag-03MSL100
Data acquisition	National Instruments	NI PXIe-4497 and NI PXI-4462

**Table 2:** The RMS of Localization Errors.

Localization Method	X-Component (cm)	Y-Component (cm)	Z-Component (cm)
Modified STAR	3.25	2.08	2.03
STAR	5.56	6.79	10.67

**Table 3:** The Computation Times of the Two Methods.

Methods	Computation Times
The traditional STAR method	3.13 (ms)
One iteration of the proposed method	0.78 (ms)
The proposed method	6.25 (ms)

**Table 4:** The Mean Value and RMS Error of the Estimated Magnetic Parameters.

Localization Method	X-Magnetic Moment (Am^2^)		Y-Magnetic Moment (Am^2^)		Z-Magnetic Moment (Am^2^)	
	Mean Value	RMS	Mean Value	RMS	Mean Value	RMS
**Modified STAR**	**0.08**	**0.23**	**0.35**	**0.18**	**−1.54**	**0.16**
**STAR**	**0.35**	**0.38**	**−0.04**	**0.48**	**−1.26**	**0.38**
